# Mechanisms of GABA_B_ receptor enhancement of extrasynaptic GABA_A_ receptor currents in cerebellar granule cells

**DOI:** 10.1038/s41598-019-53087-4

**Published:** 2019-11-13

**Authors:** Shailesh N. Khatri, Wan-Chen Wu, Ying Yang, Jason R. Pugh

**Affiliations:** 1 University of Texas Health Science Center at San Antonio, Department of Cellular and Integrative Physiology, San Antonio, TX 78229 USA; 2 Xiangya School of Medicine, Central South University, Changsha, Hunan China; 3 Center for Biomedical Neuroscience, University of Texas Health Science Center at San Antonio, San Antonio, Texas 78229 USA

**Keywords:** Ion channels in the nervous system, Neurotransmitters

## Abstract

Many neurons, including cerebellar granule cells, exhibit a tonic GABA current mediated by extrasynaptic GABA_A_ receptors. This current is a critical regulator of firing and the target of many clinically relevant compounds. Using a combination of patch clamp electrophysiology and photolytic uncaging of RuBi-GABA we show that GABA_B_ receptors are tonically active and enhance extrasynaptic GABA_A_ receptor currents in cerebellar granule cells. This enhancement is not associated with meaningful changes in GABA_A_ receptor potency, mean channel open-time, open probability, or single-channel current. However, there was a significant (~40%) decrease in the number of channels participating in the GABA uncaging current and an increase in receptor desensitization. Furthermore, we find that adenylate cyclase, PKA, CaMKII, and release of Ca^2+^ from intracellular stores are necessary for modulation of GABA_A_ receptors. Overall, this work reveals crosstalk between postsynaptic GABA_A_ and GABA_B_ receptors and identifies the signaling pathways and mechanisms involved.

## Introduction

The γ-amino butyric acid A receptors (GABA_A_Rs) are major contributors of cellular inhibition in the central nervous system. These receptors can be broadly divided into two classes based on composition and location, synaptic and extrasynaptic receptors. GABA_A_Rs are pentameric ligand gated ion channels, generally consisting of two α-subunits, two β-subunits, and either a γ- or δ-subunit. GABA_A_Rs containing a γ-subunit are targeted to the synaptic space where they are activated by synaptically released GABA and mediate phasic inhibition^1,2^. GABA_A_Rs containing a δ-subunit (δ-GABA_A_Rs), on the other hand, are found primarily in the extrasynaptic space where they experience limited exposure to synaptic GABA release and play little^3^ or no role in phasic inhibition^4^. However, δ-GABA_A_Rs show a higher affinity for GABA and can be tonically activated by ambient GABA in the extracellular space^5^. This produces a small but powerful tonic inhibitory current in cells expressing δ-subunit containing receptors, capable of dramatically influencing firing^6,7^. This has made extrasynaptic δ-GABA_A_Rs an important target for modulation. For example, the drug 4,5,6,7-Tetrahydroisothiazolo-[5,4-c]pyridine-3-ol (THIP or gaboxadol)^8^, the anesthetic propofol^9^, and ethanol^10–13^, preferentially activate δ-GABA_A_Rs. Likewise, modulation of δ-GABA_A_Rs by endogenous neurosteroids contributes to responses to stress and postpartum depression^14–16^.

Recent studies have shown that activation of GABA_B_ receptors (GABA_B_Rs) can also modulate extrasynaptic δ-GABA_A_Rs. Application of GABA_B_R agonists increases tonic GABA_A_R currents in cerebellar granule cells, dentate granule cells, and thalamic relay neurons, while GABA_B_R antagonists decrease the tonic current^17,18^. Furthermore, our studies suggest that presynaptic GABA_A_Rs may also be enhanced by activation of GABA_B_Rs in parallel fiber presynaptic terminals^19,20^. These findings open new opportunities to indirectly target tonic GABA currents mediated by δ-GABA_A_Rs. However, the signaling mechanisms linking GABA_B_R activity to δ-GABA_A_Rs and the biophysical mechanisms of GABA_A_R enhancement are not currently understood.

To address these questions, we made whole-cell patch-clamp recordings from granule cells in acute cerebellar slices, and GABA_A_R currents were evoked by photolytic release of GABA from RuBi-GABA. We found that blocking GABA_B_Rs by bath application of CGP55845 or 2hydroxy-saclofen reduced GABA_A_R currents to ~60% of control, suggesting GABA_A_R currents are constitutively enhanced by GABA_B_Rs. GABA_B_R antagonists had no effect on GABA_A_R currents in Purkinje or stellate cells, or on GABA_A_R currents evoked by synaptic GABA release in granule cells. Non-stationary fluctuation analysis suggests the change in GABA_A_R current results from a reduction in number of GABA_A_Rs activated, at least partially due to increased desensitization. Enhancement of GABA_A_R currents by GABA_B_Rs requires G-protein signaling, adenylate cyclase, PKA, and Ca^+2^/calmodulin-dependent protein kinase II (CaMKII). The reduction in GABA_A_R current by GABA_B_R antagonists was mimicked and occluded by high intracellular EGTA, suggesting changes in intracellular calcium are necessary. Inhibition of ryanodine receptors, but not L-type calcium channels, blocked the effects of GABA_B_R antagonists, suggesting GABA_B_R modulation works through release of calcium from intracellular stores.

## Results

### Extrasynaptic GABA_A_Rs are modulated by tonically active GABA_B_Rs

**Figure 1:**
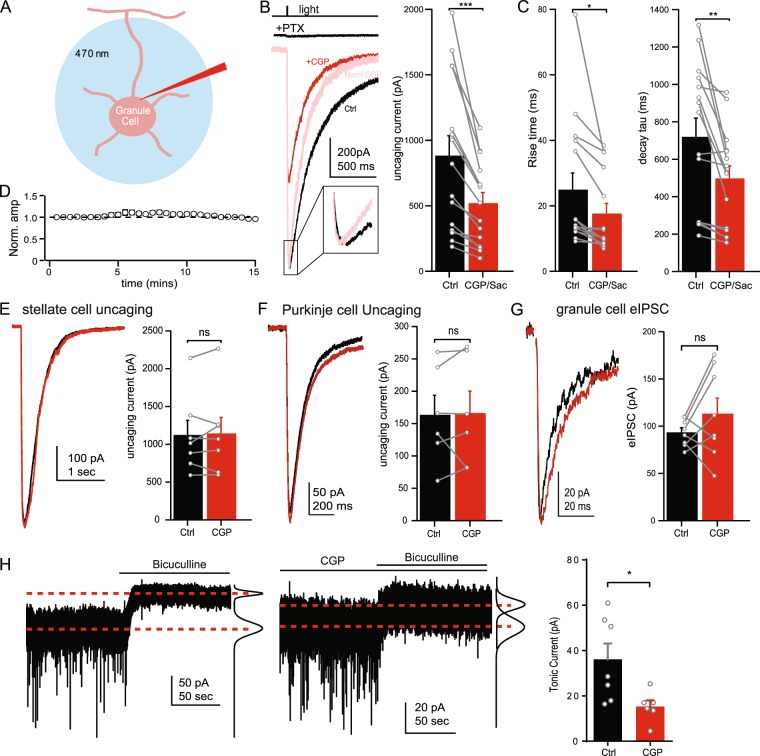
GABA_B_Rs enhance extrasynaptic GABA_A_Rs. (**A**) Schematic view of experimental design including whole-cell patch clamp recording from granule cells and uncaging of RuBi-GABA by 470 nm LED/Laser light pulse. (**B**) Left: Diagram of GABA uncaging light pulse (top) and resulting currents recorded in a granule cell in the presence of picrotoxin (+PTX), standard ACSF (black), or CGP55845 (red). The CGP trace normalized to the peak of the control trace is also displayed (pink) to show the change in decay kinetics and rise time (inset). Right: Average amplitudes of uncaging current in control (black) and in the presence of CGP55845 or saclofen (red). (**C**) Average rise time (left) and decay time constant (right) of GABA uncaging currents in control ACSF (black) or in the presence of CGP55845 or saclofen (red). (**D**) Average uncaging current amplitudes over 15 minutes in control ACSF. (**E**,**F**) Example traces (left) and average amplitudes (right) of uncaging currents in control ACSF (black) and in the presence of CGP55845 (red) in stellate cells (**E**) and Purkinje cells (**F**). (**G**) Example traces (left) and average amplitudes (right) of evoked inhibitory postsynaptic currents in control ACSF (black) and in the presence of CGP55845 (red) in granule cells. (**H**) Example traces of tonic GABA_A_ receptor currents in control ACSF (left) and in the presence of CGP (middle). Average holding current values (dashed red line) and Gaussian fits of histograms of current values (black lines) before and after bicuculin application are also shown. Right: Average tonic GABA_A_ receptor currents in control ACSF (black) and in the presence of CGP (red). Data from individual cells are plotted as connected gray markers. (*) indicates p-value ≤ 0.05, (**) indicates p-value ≤ 0.01, (***) indicates p-value ≤ *0.001, ns indicates p-value* > *0.05*.

In order to investigate the mechanisms by which GABA_B_Rs enhance GABA_A_R-mediated currents we made whole-cell patch clamp recordings from granule cells in acute cerebellar slices. GABA currents were evoked by full field uncaging of RuBi-GABA (60 μM) using a brief (5 ms) light pulse from a 470 nm LED or 473 nm laser (Fig. 1A). The resultant current is referred to as the “uncaging current”. Uncaging currents were completely blocked by application of picrotoxin (100 μM), indicating these currents are mediated by GABA_A_Rs (Fig. 1B, top). G-protein coupled inward rectifying K^+^ (GIRK) currents potentially linked to GABA_B_R activity are likely inhibited in our experiments by CsCl and QX-314 in the internal solution^21,22^. Despite the lack of GABA_B_R-mediated currents, bath application of the GABA_B_R antagonists CGP55845 (CGP, 3 μM) or saclofen (200 μM) reduced the amplitude of the uncaging current (CGP: 58.8 ± 2.7% of control, n = 9, p < 0.001; saclofen: 65.6 ± 5.7% of control, n = 6, p = 0.004; Fig. 1B), suggesting GABA_A_Rs are constitutively enhanced by GABA_B_R activity. Application of CGP or saclofen also reduced the 10–90 rise-time (24.9 ± 5.1 vs. 17.6 ± 3.1 ms, n = 15, p = 0.02) and decay-time constant (719.3 ± 101.3 vs. 497.0 ± 68.0 ms, p = 0.003) of the uncaging current (Fig. 1B,C). In the absence of CGP application, uncaging current amplitudes in granule cells were stable over 15 minutes of repeated uncaging (last response: 95.8 ± 2.7% of first response, n = 7, p = 0.18; Fig. 1D), suggesting the decrease in uncaging current amplitude is not due to rundown of the response or accumulation of GABA in the slice or bath solution. In Purkinje or stellate cells of the cerebellum, application of CGP had no effect on GABA uncaging current amplitudes (Purkinje: 103.4 ± 9.9% of control, n = 6, p = 0.83; stellate: 101.2 ± 4.6% of control, n = 7, p = 0.67; Fig. 1E,F), indicating enhancement of GABA_A_Rs is specific to granule cells. Furthermore, synaptic IPSCs evoked by electrical stimulation in granule cells were not affected by application of CGP (119.9 ± 14.7% of control, n = 8, p = 0.21; Fig. 1G), suggesting that modulation by GABA_B_Rs is specific to extrasynaptic GABA_A_Rs. This profile of activity matches δ-subunit containing GABA_A_Rs expressed in cerebellar granule cells which mediate tonic inhibition^23^. This suggests GABA_B_R modulation is specific to δ-GABA_A_Rs, as has been found previously^17,18^. However, our GABA uncaging protocol may activate both synaptic and extrasynaptic GABA_A_Rs, in order to demonstrate the same effect of CGP on isolated currents from extrasynaptic receptors, we recorded tonic GABA_A_R currents by measuring the change in the holding current following application of bicuculline (20 μM), a GABA_A_R antagonist. In the presence of CGP the tonic GABA_A_R current was decreased to 42.4% of control (n = 7, p < 0.05 unpaired t-test, Fig. 1H) similar to the reduction of uncaging currents in the presence of CGP. We therefor used GABA uncaging in further experiments to investigate the signaling mechanisms involved in extrasynaptic GABA_A_R modulation.

**Figure 2:**
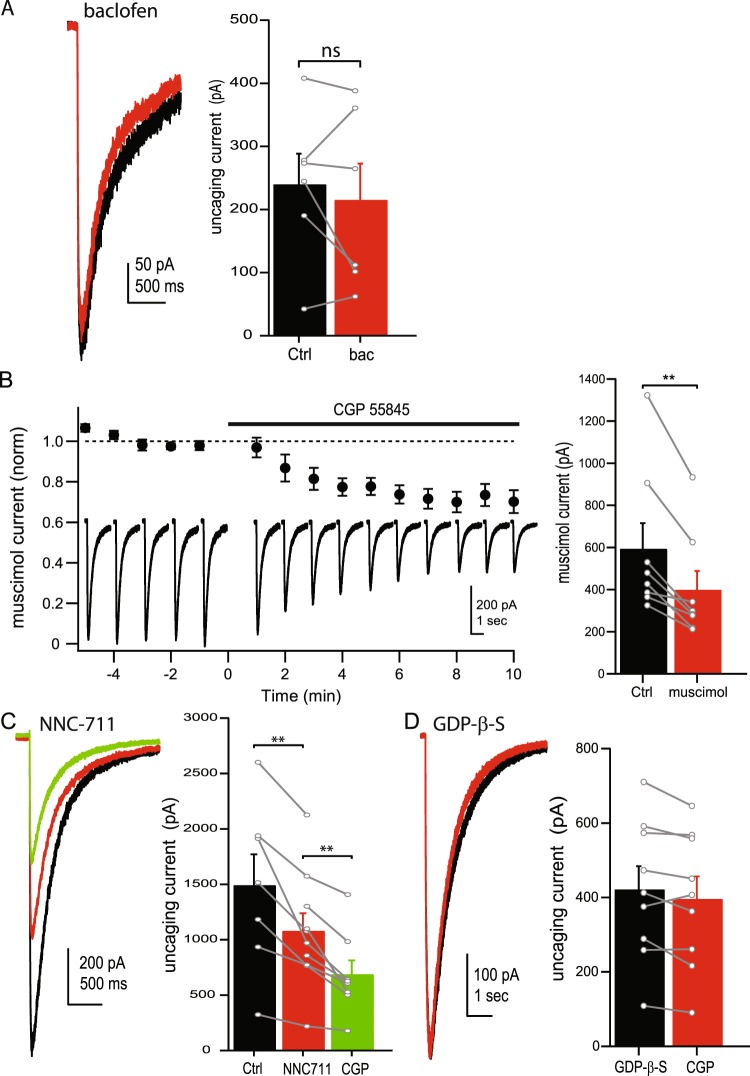
GABA_B_Rs are tonically active in granule cells. (**A**) Representative traces (left) and average amplitudes (right) of uncaging current in control ACSF (black) and in the presence of baclofen (red). (**B**) left: Average current amplitudes (circles) and example traces (inset) following pressure application of muscimol though out the time course of the experiment. CGP55845 was bath applied at time = 0. Right: Average muscimol current amplitudes in control ACSF (black) and in the presence of CGP55845 (red). (**C**) Representative traces (left) and average amplitudes (right) of uncaging currents in control ACSF (black), in the presence of NNC-711 (red), and in CGP55845 (green). (**D**) Representative traces (left) and average amplitudes (right) of uncaging currents in control ACSF (black) and in the presence of CGP55845 (red) when GDP-β-S is included in the internal solution. Data from individual cells are plotted as connected gray markers. (**) indicates p-value ≤ *0.01, ns indicates p-value* > *0.05*.

The observation that application of a GABA_B_R antagonist alone is sufficient to reduce the uncaging current suggests that at least a fraction of GABA_B_Rs are tonically activated in our experiments. This is consistent with a previous study which found that ambient GABA tonically activates GABA_B_Rs on Golgi cell terminals in the granule cell layer^24^. Furthermore, application of 100 μM baclofen, a GABA_B_R agonist, did not increase the uncaging GABA current amplitude (94.8 ± 6.2% of control, n = 6, p = 0.48; Fig. 2A), consistent with constitutive activation of GABA_B_Rs. In these experiments, GABA_B_Rs could be activated by endogenous ambient GABA in the granule cell layer^6^ or build-up of GABA in the slice due to repeated uncaging. In order to test for the presence of constitutive activation of GABA_B_Rs in the absence of repeated GABA uncaging, we activated GABA_A_Rs by local pressure application of 10 μM muscimol (a GABA_A_R specific agonist highly selective for δ-subunit GABA_A_Rs and tonic currents at low concentrations^25,26^) onto granule cells. Under these conditions we found that CGP still reduced the GABA_A_R current (67.0 ± 4.2% of control, n = 8, p = 0.002; Fig. 2B), similar to results from GABA uncaging. This suggests that GABA_B_Rs in granules cells are tonically activated by endogenous ambient GABA, not buildup of uncaged GABA in the bath solution.

In addition to granule cells, GABA_B_Rs are also expressed on the soma and axon terminals of GABAergic Golgi cells within the granule cell layer^24,27^, raising the possibility that the effects of CGP on GABA_A_R currents is mediated by network effects in the granule cell layer. A previous study found that GABA_B_Rs in Golgi cell terminals inhibit GABA release^24^, suggesting that blocking these receptors could increase GABA release in the granule cell layer. Moreover, in cell attached recordings we found that application of CGP increased the spontaneous firing rate of Golgi cells (226.4 ± 39.81% of control, p = 0.02, n = 3), which is also expected to increase GABA release. This raises the possibility that CGP acts on GABA_A_Rs through increasing the ambient extracellular GABA concentration in the granule cell layer. This could reduce the uncaging GABA current through either increasing desensitization of GABA_A_Rs^5^ or increasing the fraction of receptors tonically activated (and reducing the fraction of receptors available to be activated by GABA uncaging). We tested this possibility by applying 10 µM NNC-711, a GABA transporter blocker, to raise the ambient GABA level. In four out of four cells NNC-711 increased the tonic GABA current (data not shown), confirming an increase in ambient GABA. NNC-711 reduced the uncaging current amplitude (71.4 ± 4.4% of control, n = 7, p = 0.007), but subsequent application of CGP was still able to reduce the uncaging current amplitude further (72.2 ± 4.4% of NNC-711, n = 7, p = 0.002, Fig. 2C). These data indicate that increasing the ambient GABA level can alter GABA uncaging currents, but this does not occlude the reduction in uncaging current amplitude by CGP. In a second set of experiment, GDP-β-S was included in the internal solution to inhibit G-protein coupled signaling in the patched cell. In this case, CGP had little effect on the uncaging current amplitude (92.8 ± 3.3% of control, n = 9, p = 0.05; Fig. 2D), suggesting that GABA_B_R signaling within the patched granule cell is necessary for enhancement of extrasynaptic GABA_A_Rs and ruling out network effects as a likely mechanism. This finding also confirms that CGP acts through a G-protein coupled receptors (GPCR) rather than binding to or altering GABA_A_Rs directly.

### GABA_B_ receptors increase the number of available GABA_A_ receptors

While previous studies and our data show that GABA_B_Rs enhance extrasynaptic GABA_A_Rs^17,18^, the biophysical mechanisms of this enhancement have not been investigated. We tested several potential mechanisms that could alter GABA_A_R currents, including changes in GABA potency, desensitization, single channel open time, single channel current, open probability, and number of receptors available.

**Figure 3:**
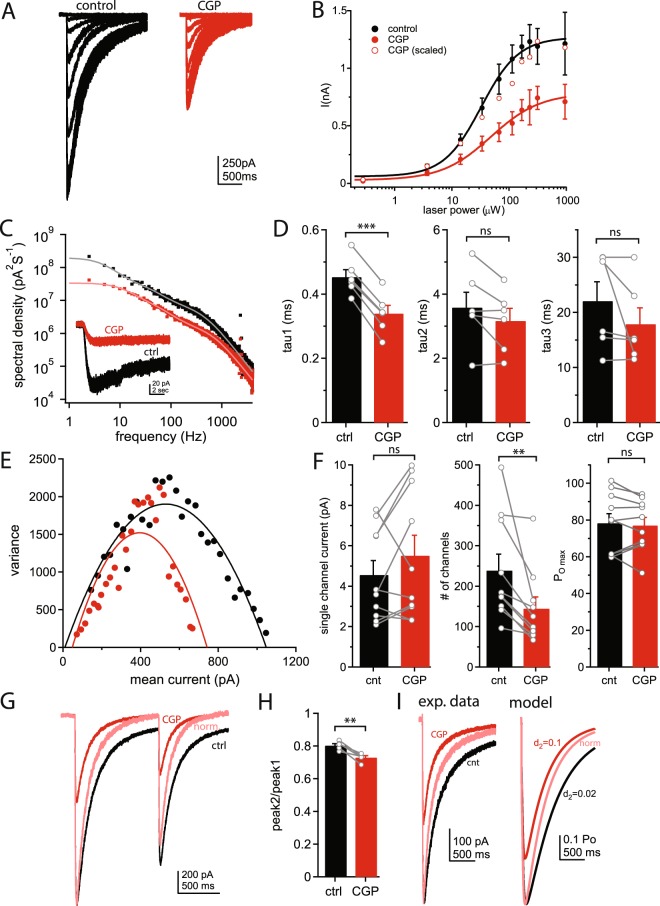
GABA_B_R inhibition reduces the number of active GABA_A_Rs. (**A**) Representative traces of uncaging currents elicited by varying the uncaging laser intensity in control ACSF (left) and in the presence of CGP55845 (right). (**B**) Dose response curve showing the relationship between uncaging laser power and uncaging current amplitude in control ACSF (black) and in the presence of CGP55845 (red). Data are fit with Hill equation (lines). The CGP data normalized to the maximum current in control is also shown (open circles). (**C**) Example current traces (inset) and power spectral density plot of uncaging currents in control ACSF (black) and in the presence of CGP55845 (red). (**D**) Average values of fast (left) and slow (middle, right) time-constants determined by power spectral analysis in control ACSF (black) and in the presence of CGP55845 (red). (**E**) Example plot of non-stationary fluctuation analysis. Plot shows mean current amplitudes plotted against the variance of the current in control ACSF (black) and in the presence of CGP55845 (red) for a single cell. (**F**) Average single channel current (left), number of channels participating in the current (middle), and channel open probability (P_O max_; right) in control ACSF (black) and in the presence of CGO55845 (red). (**G**) Representative uncaging current traces in control ACSF (black) on in the presence of CGP (red) following a pair of uncaging light pulses. The CGP trace normalized to the first peak of the control trace is also shown (pink) to demonstrate the change in paired-pulse ratio. (**H**) Average paired-pulse ratio in control ACSF (black) or in the presence of CGP55845 (red). (**I**) Left: Example traces of GABA uncaging currents in control ACSF (black) or in the presence of CGP (red). The CGP trace normalized to the peak of the control trace is also shown (pink) for comparison of kinetics. Right: Simulated GABA_A_R gating showing responses with relatively slow (black) and fast (red) desensitization. The fast desensitization trace normalized to the peak of the slow desensitization trace is also shown (pink) for comparison. *Control data (black) in (A) and (B) were previously published in Khatri et al*.^20^. Data from individual cells are plotted as connected gray markers. (**) indicates p-value ≤ 0.01, (***) indicates p-value ≤ *0.001, ns indicates p-value* > *0.05*.

To test whether GABA_B_R activity alters the GABA potency of GABA_A_Rs in granule cells we used a range of blue laser intensities to uncage increasing concentrations of GABA and recorded the resulting currents (Fig. 3A). From this data, we created a dose-response curve of GABA_A_Rs in control solutions and in the presence of CGP. We found that the uncaging current was reduced to ~60% of control for each laser intensity tested, with no right or leftward shift in the curve (Fig. 3B). In fact, when the responses in CGP were scaled to the maximum response in control, the curves show significant overlap (Fig. 3B, open circles), suggesting GABA_B_R activity does not change the GABA potency of GABA_A_Rs.

We also measured the mean-open times of GABA_A_Rs using power spectra analysis of GABA currents during long (12 sec) pressure applications of GABA in control ACSF and in the presence of CGP (see methods; Fig. 3C, inset). From these data we constructed plots of spectral density versus frequency for each cell, which were fit with Lorentzian functions. From these fits we were able to estimate the mean channel open times for each cell. We found a small, but significant, reduction in the fast-open time in the presence of CGP (0.45 ± 0.02 ms vs 0.33 ± 0.03 ms, n = 6, p < 0.001; Fig. 3D), and no change in the slower open times (p > 0.05). Baclofen had no effect on the fast open time (0.56 ± 0.12 ms vs 0.48 ± 0.15 ms, n = 5, p = 0.66). The faster open-time may contribute to the more rapid rise-time and decay time of the GABA current, but likely does not account for the relatively large (~40%) reduction in the macroscopic current.

We then used non-stationary fluctuation analysis^28–30^ of currents evoked by uncaging GABA to discern whether CGP induced a change in the single-channel conductance, open probability, or number of available GABA_A_Rs. For each cell we measured the current amplitude and variance during the decay phase of the current, from which a plot of variance versus the mean amplitude of the current was constructed and fit with a binomial model (see methods). From these fits we estimated the single channel conductance, open probability, and number of channels participating in the current for each cell before and after application of CGP (Fig. 3E). Application of CGP did not change the single channel conductance (118.8 ± 12.4%, n = 12, p = 0.24) or maximum open probability (P_O max_; 99.3 ± 2.9%, n = 12, p = 0.56) of GABA_A_Rs, however, the number of channels participating in the current was reduced to 61.8 ± 5.9% of control (n = 12, p = 0.005; Fig. 3F). This matches closely the reduction in the macroscopic uncaging current observed (~60% of control). The decrease in number of channels participating in the current could represent removal of GABA_A_Rs from the membrane or accumulation of receptors into long-lasting desensitized states^31^.

Previous investigations of desensitization of extrasynaptic/δ-subunit containing GABA_A_R have produced conflicting results, with some studies showing little or no desensitization^32,33^ and other showing moderate to profound desensitization^2,5,34^. To test for desensitization in our preparation and whether it is altered by GABA_B_R activity, we delivered a pair of uncaging light pulses (1 second interval) and measured the peak current amplitudes. The second peak (measured from the baseline prior to the first peak) was consistently smaller than the first peak, indicating desensitization of GABA_A_Rs following the first GABA application (peak2/peak1: 0.80 ± 0.01, n = 5, p = 0.004; Fig. 3G). Bath application of CGP, in addition to reducing both peak current amplitudes (p = 0.007), also significantly reduced the ratio of the second peak to the first (peak2/peak1: 0.80 ± 0.01 versus 0.73 ± 0.01, p = 0.005; Fig. 3H), indicating greater desensitization. This suggests that increased desensitization contributes to the decreased GABA_A_R current and kinetics. However, these changes in desensitization are likely not sufficient to completely account for the large (~40%) decrease in the number of GABA_A_Rs participating in the GABA current following CGP application, suggesting changes in receptor trafficking and internalization are also involved.

We then tested whether a simple change in the rate of desensitization could account for the changes observed following CGP application using a computer model of GABA_A_R gating based on the kinetic scheme first described by Jones and Westbrook^35^. The transition rates in the model were first optimized to fit the relatively slow rise-time and decay kinetics of GABA_A_R currents evoked by GABA uncaging in cerebellar granule cells (Fig. 3I, Table 1). In the model receptors were activated by a 5 ms square pulse of 60 μM GABA followed by an exponential decay to mimic the time course and relatively low concentrations of GABA likely to result from our uncaging protocol. In order to test the effects of increasing desensitization, we increased the transition rate to the doubly bound desensitized state 5-fold. We found that this change alone was sufficient to closely replicate the observed effects of CGP on GABA_A_R currents, including reduced peak current, reduced rise-time of the current, and reduced rate of decay (Fig. 3I, Table 1). Furthermore, both the experimental and model currents were well fit by single exponential curves. The faster current decay in the model is surprising given that previous modeling of GABA_A_Rs have found that increasing desensitization results in slower, bi-exponential current decay^35,36^ due to reopening of desensitized receptors before unbinding GABA. This difference in our results appears to be due primarily to the relatively slow back rate from the doubly bound desensitized state in our model, and may reflect unique properties of the extrasynaptic (δ-subunit containing) GABA_A_Rs modeled here as opposed to synaptic (γ-subunit containing) receptors. While this modelling data replicates the experimental data in many aspects, this does not necessarily mean that same process takes place in granule cells. However, this does provide theoretical evidence that it is possible for an increase in the rate of desensitization to produce faster macroscopic current kinetics.

**Table 1:** Kinetic parameters of experimentally recorded and simulated GABA_A_R currents.

	Rise-time (ms)	Tau decay (ms)	Peak (CGP/cnt)
Expmt. (cnt)	24.9 ± 5.1	719.3 ± 101.3	
Expmt. (CGP)	17.6 ± 3.1	497.0 ± 68.0	61.6 ± 3.9
Model (d_2_ = 0.02)	46.1	677.9	
Model (d_2_ = 0.1)	38.7	439.1	75.5

### Signaling mechanisms of GABA_A_ receptor enhancement

**Figure 4:**
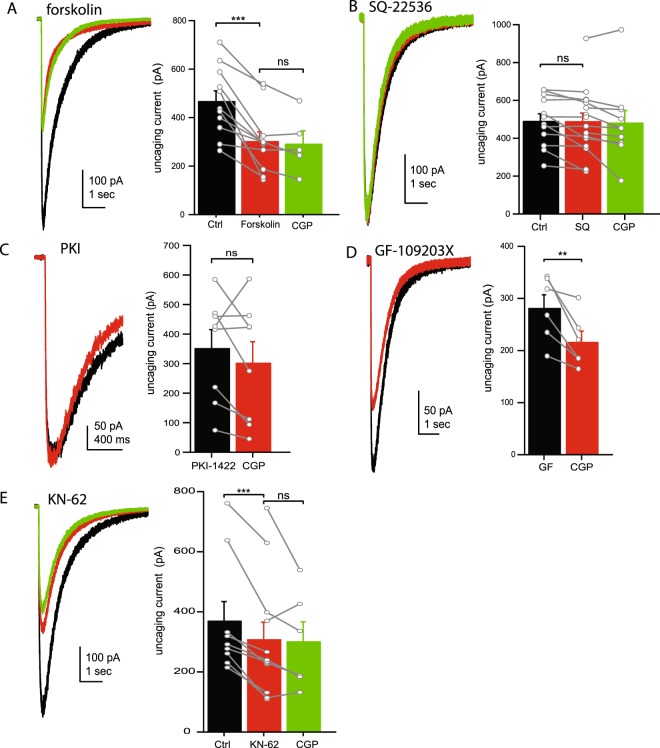
Intracellular kinase dependent enhancement of GABA_A_Rs. (**A**) Representative uncaging current traces (left) and average current amplitudes (right) in control ACSF (black), in the presence of the adenylate cyclase activator, forskolin (red) and following the addition of CGP55845 (green). (**B**) Representative uncaging current traces (left) and average current amplitudes (right) in control ACSF (black), in the presence of the adenylate cyclase inhibitor, SQ-22536 (red) and in the presence of CGP55845 (green). (**C**) Representative uncaging current traces (left) and average current amplitudes (right) in control ACSF (black) and in the presence of CGP55845 (red) when the PKA inhibitor, PKI 14–22, is included in the internal solution. (**D**) Representative uncaging current traces (left) and average current amplitudes (right) in control ACSF (black) and in the presence of CGP55845 (red) when the PKC inhibitor GF-109203X is included in the bath solution. (**E**) Representative uncaging current traces (left) and average current amplitudes (right) in control ACSF (black), in the presence of the CaMKII inhibitor, KN-62 (red) and following the addition of CGP55845 (green). Data from individual cells are plotted as connected gray markers. (**) indicates p-value ≤ 0.01, (***) indicates p-value ≤ *0.001, ns indicates p-value* > *0.05*.

The G_i/o_ coupled GPCRs, like GABA_B_Rs, have been shown to inhibit adenylate cyclase which produces cAMP, a common second messenger molecule^37,38^. To test whether inhibition of adenylate cyclase is required for enhancement of GABA_A_Rs we measured uncaging currents in granule cells before and after application of forskolin (an adenylate cyclase activator) or SQ-22536 (an adenylate cyclase inhibitor). We found that forskolin (10 μM), reduced the uncaging current amplitude (65.3 ± 5.8% of control, n = 11, p < 0.001; Fig. 4A). Furthermore, application of CGP in the presence of forskolin had no further effect on the amplitude of uncaging currents (89.9 ± 4.9% of control, n = 5, p = 0.11), indicating activation of adenylate cyclase mimics and occludes the effect of CGP. This is consistent with previous observations and our model in which adenylate cyclase activity is disinhibited by application of CGP^17,39,40^. In the presence of the adenylate cyclase inhibitor, SQ-22536 (100 μM), application of CGP had only a minimal effect on the uncaging current amplitude (89.1 ± 5.2% of control, n = 9, p = 0.04; Fig. 4B), suggesting activation (or disinhibition) of adenylate cyclase is necessary for the reduction in GABA current by CGP. Together, these data suggest that adenylate cyclase is normally inhibited by tonic GABA_B_R activity, but becomes disinhibited/activated when GABA_B_Rs are blocked by CGP.

How does activation of adenylate cyclase result in reduced GABA_A_R currents? Previous reports have shown that several conserved intracellular serine or tyrosine residues on GABA_A_R subunits are phosphorylation sites for kinases, including PKA, PKC, and CaMKII^41–44^. Phosphorylation of these residues maintains surface expression of GABA_A_Rs, whereas, dephosphorylation of these subunits triggers receptor internalization^45,46^. We first tested whether PKA is required for GABA_A_R enhancement by GABA_B_Rs, as it is a primary target of adenylate cyclase/cAMP activity. To block PKA activity we included the PKA inhibitory peptide PKI 14–22 (1 μM) in the intracellular pipette solution. In the presence of this inhibitor the effects of CGP were highly variable. Overall, the change in uncaging current amplitude was not significant (80.1 ± 10.9% of control, n = 8, p = 0.26; Fig. 4C), suggesting PKA activity may be required for the effect of CGP on the GABA current. This is consistent with previous findings in many cells that PKA is normally inhibited by G_i/o_ GPCRs (including GABA_B_Rs) and application of CGP results in activation/disinhibition of PKA^37,38^.

In order to identify other kinases involved in GABA_B_R-mediated modulation of the uncaging current, we next tested CaMKII and PKC. The inclusion of the PKC inhibitor GF-109203 × (1 µM) in the bath solution did not inhibit the effects of CGP on the uncaging current (77.7 ± 4.7% of control, n = 6, p = 0.01; Fig. 4D). However, the inclusion of the CaMKII inhibitor KN-62 (2 μM) in the bath solution abolished the effects of CGP on the uncaging current (90.9 ± 7.9 of control, n = 6, p = 0.24, Fig. 4E). Furthermore, application of KN-62 alone was sufficient to reduce the uncaging current (68.5 ± 5.3% of control, n = 9; p < 0.001), indicating KN-62 mimics and occludes the effects of CGP. These data suggest that CGP reduces the uncaging current through inhibition of CaMKII.

**Figure 5:**
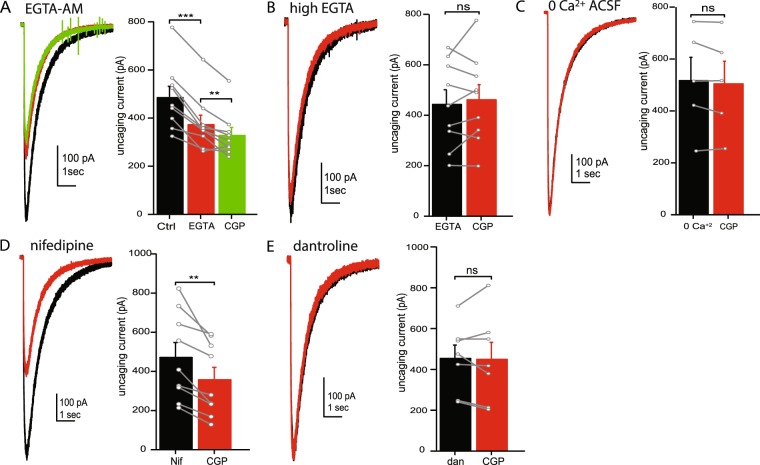
Intracellular calcium dependent enhancement of GABA_A_Rs. (**A**) Representative uncaging current traces (left) and average current amplitudes (right) in control ACSF (black), in the presence of the EGTA-AM (red) and following the addition of CGP55845 (green). (**B**) Representative uncaging current traces (left) and average current amplitudes (right) in control ACSF (black) and in the presence of CGP55845 (red) when high (10 mM) EGTA is included in the internal solution. (**C**) Representative uncaging current traces (left) and average current amplitudes (right) in 0 calcium ACSF (black) and following application of CGP55845 (red). (**D**) Representative uncaging current traces (left) and average current amplitudes (right) in the presence of nifedipine (black), and following addition of CGP55845 (red). (**E**) Representative uncaging current traces (left) and average current amplitudes (right) in the presence of dantrolene (black) and following addition of CGP55845 (red). Data from individual cells are plotted as connected gray markers. (**) indicates p-value ≤ 0.01, (***) indicates p-value ≤ *0.001, ns indicates p-value* > *0.05*.

CaMKII is regulated by intracellular calcium^47,48^, raising the possibility that the pathway from GABA_B_Rs to GABA_A_Rs involves changes in intracellular calcium. To test this we bath applied the cell permeable calcium chelator EGTA-AM, which becomes enriched inside the cell due to cleavage of the AM group. Application of 20 µM EGTA-AM alone was sufficient to reduce uncaging current amplitudes (77.21 ± 2.86% of control, n = 9, p < 0.001; Fig. 5A). In the presence of EGTA-AM, further application of CGP had only a small effect on the current amplitude (88.92 ± 3.33% of EGTA-AM, n = 9, p = 0.007), possibly due to continued accumulation of EGTA inside the cell. When using a high EGTA (10 mM) internal solution, CGP application had no effect on GABA_A_R current amplitudes (105.97% ± 5.52 of control, n = 9, p = 0.43; Fig. 5B). Furthermore, in slices bathed in calcium-free ACSF, application of CGP did not alter the uncaging current amplitude (98.1 ± 2.04% of control, n = 5, p = 0.26; Fig. 5C). These data suggest that changes in intracellular Ca^+2^ are required for enhancement of GABA_A_Rs by GABA_B_Rs.

Two primary sources of intracellular calcium are voltage-gated calcium channels and calcium release from intracellular stores. We first tested whether Ca^+2^ influx through L-type calcium channels is necessary for the CGP effect, as these channels make up approximately 90% of voltage-gated calcium channels (VGCC) in granule cell bodies^49^. In the presence of nifedipine (an L-type channel blocker), we still observed a significant reduction of the uncaging current amplitude following application of CGP (74.6 ± 4.6% of nifedipine, n = 9, p = 0.003), suggesting L-type calcium channels are not necessary for the effects of CGP. We then tested whether calcium release from intracellular stores is necessary for the effects of CGP. In slices preincubated in dantrolene (10 µM), uncaging currents were not altered by application of CGP (95.83 ± 4.8% of control, n = 7, p = 0.85; Fig. 5F), confirming that ryanodine receptor-mediated release of calcium from intracellular stores is required for the effects of CGP on GABA_A_Rs. These data suggest that increases in intracellular calcium from intracellular stores can activate CaMKII and enhance GABA_A_R-mediated currents.

## Discussion

We find that GABA_B_Rs in cerebellar granule cells are tonically active and selectively enhance currents through extrasynaptic (presumably δ-subunit containing) GABA_A_Rs. Blocking GABA_B_Rs reduces the number of GABA_A_ channels activated by GABA, due to changes in desensitization and trafficking of receptors. Further, we find that GABA_B_R-mediated enhancement of GABA_A_Rs is dependent on signaling pathways including adenylate cyclase, PKA, CaMKII, and release of calcium from intracellular stores. Identification of the biophysical mechanisms and signaling pathway involved in regulation of tonic GABA_A_R currents will be important for understanding how ambient GABA and tonic inhibition in the granule cell layer regulates activity at the input layer of the cerebellum. Furthermore, the same mechanisms and pathways are likely employed in other cell types expressing extrasynaptic δ-GABA_A_Rs, including thalamic relay neurons and dentate granule cells of the hippocampus. Our recent studies show that presynaptic GABA_B_Rs can also modulate GABA_A_Rs expressed in the presynaptic terminals of parallel fibers^19,20^. Many other presynaptic terminals throughout the central nervous system also co-express GABA_A_ and GABA_B_Rs, raising the possibility that GABA_B_R-mediated enhancement of GABA_A_Rs is a widespread mechanism of synaptic modulation in presynaptic terminals.

### Tonic GABA_B_ receptor activity

The granule cell layer maintains basal levels of ambient GABA providing an additional layer of inhibition through extrasynaptic GABA_A_Rs^6,50,51^. While many studies have shown that ambient GABA tonically activates GABA_A_Rs in granule cells, tonic activation of GABA_B_Rs has received relatively little attention. Estimates of the GABA EC_50_ for GABA_B_Rs can vary widely, however, several reports put this value in the low nanomolar range (5–20 nM^38,52,53^), well within the range for activation by ambient GABA (150–250 nM^5,54^). We have recently shown that GABA_B_Rs in the axons of granule cells respond to lower concentrations of GABA than GABA_A_Rs^19^, suggesting GABA_B_Rs may also be tonically activated in granule cells. This possibility is further supported by findings that GABA_B_Rs expressed on synaptic terminals of Golgi cells are tonically activated^24^. In this study, we find that application of a GABA_B_R antagonist alone is sufficient to reduce tonic GABA/muscimol evoked GABA_A_R currents (Figs 1H, 2B), suggesting GABA_B_Rs are constitutively activated. In addition, application of a GABA_B_R agonist, baclofen, does not alter the uncaging GABA current, consistent with prior activation of GABA_B_Rs by ambient GABA. These results differ from an earlier study which showed that baclofen increases the tonic current in cerebellar granule cells while CGP slightly decreases the tonic current^17^. These conflicting results may be due to the fact that the previous study used tetrodotoxin to block neuronal activity, shown to lower the level of ambient GABA in the granule cell layer at this age^6,55^, and limiting tonic GABA_B_R activity.

### Biophysical mechanisms of GABA_A_ receptor enhancement

There are several biophysical mechanisms that can account for changes in GABA_A_R-mediated currents, including changes in chloride reversal potential, GABA potency, channel open time, desensitization, single channel conductance, channel open probability, or the number of channels in the membrane. Recent work has shown that GABA_B_Rs can regulate the chloride transporter KCC2 altering the chloride reversal potential in neurons^56^. However, this is unlikely to account for the change in uncaging current in our experiments as the chloride reversal potential is determined by the relative chloride concentrations in the internal and bath solutions. We did not find changes in GABA potency (Fig. 3B), single channel conductance, or open probability (Fig. 3F). However, our data does suggest changes in channel gating contribute to the effects of blocking GABA_B_Rs. We observed a consistent decrease in the fast mean open time of the channel (Fig. 3D) and increased desensitization following GABA application (Fig. 3G), which may account for the increased rise-time and decay kinetics of the macroscopic GABA current. In a computer model of GABA_A_R gating we tested whether increasing the rate of desensitization could account for these changes and found that this change alone was sufficient to reduce the peak current amplitude, the rise-time, and the decay time. However, the fast open time account for only 3–5% of current fluctuations and the change in desensitized receptors was relatively small (5–10%), suggesting other mechanisms may also contribute to the relatively large change in the uncaging current. Using non-stationary fluctuation analysis we found that the number of channels participating in the GABA current was significantly reduced by CGP application (Fig. 3F). This decrease in available channels can be explained, at least in part, by increased accumulation of receptors into long-lasting desensitized states in response to low levels of ambient GABA, as has been reported at other GABA_A_Rs^5,31^. The literature on desensitization of δ-subunit containing GABA_A_Rs has been mixed. Initial reports suggest these receptors show little or no desensitization^32^. More recent studies using α_6_β_2/3_δ receptors (the subunit composition thought to mediate the tonic current in cerebellar granule cells) or extrasynaptic receptors in granule cells suggest moderate to profound desensitization of these receptors^2,5,33,34^. However, changes in receptor trafficking and surface expression may also be necessary to account for the 40–50% reduction of the macroscopic GABA_A_R current. We therefor argue that GABA_B_R activity/blockade also alters trafficking and surface expression of GABA_A_Rs. Previous studies provide ample evidence that phosphorylation of GABA_A_Rs by common kinases (CaMKII, PKA, PKC) can alter GABA_A_R trafficking and surface expression^41–44^.

### Signaling mechanisms of GABA_A_R enhancement

The primary signaling pathways of GABA_B_Rs, like all G_i/o_ coupled GPCRs, is through inhibition of adenylate cyclase and reduction of cAMP production^37,38^. In our experiments, GABA_B_Rs are tonically activated, thus application of the GABA_B_R antagonist, CGP-55845, would disinhibit (or activate) adenylate cyclase and cAMP production. Consistent with this, we find that forskolin, an activator of adenylate cyclase, mimics and occludes the effects of CGP. Further, in the presence of SQ 22536, an adenylate cyclase inhibitor, CGP has no effect on the GABA_A_R current because there is no disinhibition of adenylate cyclase in this case. One of the main targets of cAMP in intracellular signaling cascade is PKA, thus disinhibition (or activation) of adenylate cyclase/cAMP production would activate PKA^37,38^. Consistent with this, we find that if activation of PKA is prevented by including a PKA inhibitory peptide in the intracellular solution, application of CGP does not significantly reduce the GABA_A_R current. This suggests activation of PKA is required for the CGP effect, and is consistent with earlier findings that PKA activity decreases tonic GABA_A_R currents in thalamo-cortical neurons^17^. However, phosphorylation of GABA_A_Rs by PKA can either increase or decrease surface expression of receptors depending on β subunit expression (β_1_ subunit containing receptor expression is decreased while β3 subunit containing receptor expression is increased)^43,44,57^. Cerebellar granule cells primarily express β_2_ and β_3_, though β_1_ expression is also present at lower levels^58,59^. This suggests phosphorylation of GABA_A_Rs by PKA would primarily increase GABA_A_R expression, while we find that disinhibition/activation of PKA results in reduced GABA_A_R-mediated currents. It is therefore unlikely that PKA activity is directly involved in phosphorylating GABA_A_Rs. Rather, other signaling molecules/kinases must be involved.

Several studies have shown that CaMKII and PKC can also phosphorylate GABA_A_Rs and increase cell surface expression^41,60–62^. In cell expression system or in cerebellar granule cells, activation of CaMKII enhanced surface expression of GABA_A_Rs containing a β3 subunit but not receptors containing a β2 subunit^47^. Furthermore, the expression of β3 subunits may contribute to receptor trafficking and the cellular location of receptor expression^63^. In our experiments blocking CaMKII, but not PKC, abolished the effect of CGP application on GABA_A_R currents. In fact, application of KN-62 (a CaMKII inhibitor) alone was sufficient to reduce the GABA_A_R current, suggesting that CGP acts through inhibition of CaMKII. This raises the possibility that GABA_B_R modulation acts through CaMKII dependent phosphorylation of extrasynaptic, but not synaptic receptors, based on β subunit expression.

CaMKII activity is highly dependent on calcium, raising the possibility that changes in intracellular calcium levels are involved in the CGP effect. Indeed, we find that the effects of CGP are absent in the presence of high EGTA or following removal of extracellular calcium (Fig. 5B,C). There are two primary sources of intracellular calcium in neurons, influx of calcium through voltage-gated calcium channels and release of calcium from intracellular stores. Inhibition of calcium release from intracellular stores, but not inhibition of L-type calcium channels (which constitute ~90% of voltage-gated calcium channels in granule cells^49^), abolished the effects of CGP (Fig. 5F), suggesting calcium release from intracellular stores is more important than calcium influx for this effect. However, the observation that the CGP effect is abolished by removal of calcium from the bath solution suggests influx of calcium may also be necessary. It is tempting to speculate that calcium influx (through channels other than L-type channels) induces calcium release from intracellular stores. However, mature granule cells show little evidence of calcium induced calcium release^64–66^, suggesting this is not the case. Another possibility is that bathing the slice in 0 Ca^2+^ aCSF results in a slow depletion of intracellular stores, and subsequent loss of the CGP effect.

The requirement of release of calcium from intracellular stores would seem to implicate G_q_ coupled receptors, not G_i/o_ coupled receptors such as the GABA_B_R, which signal primarily through adenylate cyclase and cAMP. However, a number of previous studies have also observed a rise in calcium from intracellular stores as a result of GABA_B_R activation in a variety of cells^67–69^ including cerebellar granule cells^70^. While the entire signaling pathway remains unclear, it has been shown to depend on signaling through IP3 and IP3 receptors^71^. One possible mechanism is through activity of the G_βγ_ subunit, which has been shown to stimulate PLC_β_1–3^72^ and/or formation of IP3^73–75^. This raises the possibility that GABA_B_Rs signal through PKA with the G_α_ subunit and stimulate release of calcium from intracellular stores with the G_βγ_ subunit. While this possibility is consistent with our data, we do not have any direct evidence of GABA_B_R-mediated activation of PLC or formation of IP3.

**Figure 6:**
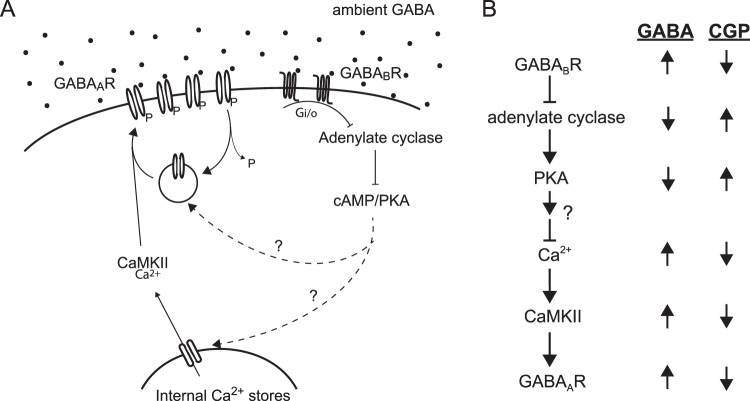
GABA_B_R signaling model. (**A**) Diagram of the signaling mechanism linking GABA_B_R activation to enhancement of extrasynaptic GABA_A_Rs. (**B**) Chart indicating predicted changes at each step in the signaling pathway when GABA_B_Rs are activated (GABA) or inhibited (CGP).

Based on our data we propose a signaling pathway following GABA_B_R activation involving inhibition of adenylate cyclase and PKA (Fig. 6). This directly or indirectly produces a downstream increase in calcium release from intracellular stores and activation of CaMKII. Finally, CaMKII phosphorylates extrasynaptic GABA_A_Rs resulting in decreased desensitization and/or insertion of receptors into the surface membrane.

### Physiological relevance

The granule cell layer is the primary input layer of the cerebellum, where granule cells integrate and process information received from across many brain regions^76^. Spike processing in granule cells is influenced by phasic inhibition from Golgi cell terminals, and tonic inhibition mediated by extrasynaptic GABA_A_Rs. It has been estimated that tonic inhibition accounts for 25–75% of chloride influx in granule cells^77^ and regulates the gain of the input/output function of these cells^78^. Previous work has shown that the level of ambient GABA in the cerebellum can be modulated, affecting granule cell firing and cerebellar function. For example, neuroinflammation in the cerebellum has been shown to increase ambient GABA, resulting in impaired motor coordination^79^. Our data suggest that GABA_B_R enhancement of GABA_A_Rs may amplify the effects of ambient GABA on granule cell firing. Low levels of ambient GABA likely produce little activation of GABA_A_ or GABA_B_Rs, resulting in little or no tonic GABA_A_R current. However, as the ambient GABA concentration increases the tonic GABA_A_R current is expected to increase due to both direct activation of GABA_A_Rs and due to increased activation of GABA_B_Rs and the resulting enhancement of extrasynaptic GABA_A_Rs. This creates a much steeper response of GABA_A_Rs to changes in ambient GABA than would be expected from the biophysical properties of the GABA_A_Rs alone and increases the sensitivity of granule cells to changes in ambient GABA.

### Future directions

Cerebellar granule cells express a range of G-protein coupled receptors including, GABA_B_Rs^80^, adenosine A1 receptors^81^, orexin receptors^82^, and glutamate receptors (mGluRs)^83^. This raises the question of whether modulation of extrasynaptic GABA_A_Rs is specific to GABA_B_Rs in these cells or if other types of G-protein coupled receptors can also activate/inhibit this pathway. It was recently shown that orexin receptors (OX1 and OX2) are expressed in granule cells and their activation decreases GABA_A_R-mediated currents in a calcium dependent manner^82^. This suggests that orexin receptors may employ the same mechanisms as GABA_B_Rs to modulate GABA_A_R function, though in the opposite direction. The opposite effect of orexin receptors on GABA_A_R-mediated currents may be accounted for by the fact that orexin receptors couple to G_s_ subunits (in addition to G_i/o_ and G_q_)^84^ which generally activate adenylate cyclase, unlike G_i/o_ subunits which inhibit adenylate cyclase. These data suggests that orexin receptors, and possibly other G_s_ coupled receptors, may regulate tonic GABA_A_R-mediated currents in opposition to GABA_B_Rs using the same signaling pathways. Future experiments will be required to determine if other G-protein coupled receptors, besides GABA_B_Rs, also employ the signaling pathway/biophysical mechanisms to regulate GABA_A_Rs.

Our results indicate that only extrasynaptic receptors in granule cells are modulated by GABA_B_R activity. This result is consistent with δ-subunit expression patterns, but does not directly implicate these receptors. It remains possible that the δ-subunit itself is not necessary for this form of modulation and other extrasynaptic receptors lacking the δ-subunit can also be modulated by this mechanism. In the future these questions can be addressed using cell-type specific δ-subunit knock-out mice.

## Experimental Procedures

### Animals

All experimental procedures involving animals were approved by the Institutional Animal Care and Use Committee at UT Health San Antonio and followed the guidelines of the *National Institutes of Health’s Guide for the Care and Use of Laboratory Animals*. Male and female C57BL/6 mice (Charles River, MA) 14–30 days old were used for all experiments. Animals were kept on a 12/12 hour light dark cycle with *ad libitum* access to food and water.

### Slice preparation

Mice were deeply anesthetized with isoflurane and the cerebellum was rapidly dissected following decapitation and placed in ice-cold oxygenated ACSF containing the following (in mM): 119 NaCl, 26.2 NaHCO_3_, 2.5 KCl, 1.0 NaH_2_PO_4_, 11 glucose, 2 CaCl_2_, 1.3 MgCl_2_ as described previously^19^. Parasagittal slices (250–300 µm) were cut from the vermis of the cerebellum using a vibratome (Leica Biosystems, IL) and then incubated at 34 °C for 30 min, after which they were used for electrophysiological recordings at room temperature.

### Electrophysiology experiments

For recording, slices were gently place in a chamber that was perfused with room temperature ACSF containing the following (in mM): 119 NaCl, 26.2 NaHCO_3_, 2.5 KCl, 1.0 NaH_2_PO_4_, 11 glucose, 2 CaCl_2_, 1.3 MgCl_2_ (flow rate of ~2 ml/min) housed in a SliceScope Pro upright microscope (Scientifica Instruments, UK.). For experiments on somatodendritic GABA currents, whole cell patch clamp recordings were made from granule cells, Purkinje cells, and stellate cells with the following internal (in mM): 135 CsCl, 10 HEPES, 4 MgCl_2_, 5 EGTA, 4 Na-ATP, 0.5 Na-GTP, 2 QX-314. Additional drugs were included in the internal solution as indicated in the text. The pH of the internal solution was adjusted to 7.2–7.4 using CsOH and the osmolarity was 280–300 mosmol. Cells were patched using 4–6 MΩs borosilicate glass pipettes (Sutter instruments) that were pulled on Sutter pipette puller (Model P-100, Sutter instruments, CA, USA). Electrophysiological currents were recorded with a Multiclamp 700B amplifier (Molecular Devices, CA), filtered at 5 kHz and digitized at 50 kHz. Data were collected using pCLAMP software (Molecular Devices). For GABA uncaging experiments, 10 ml of ACSF containing 10 µM NBQX (Tocris, MN) and 60 μM RuBi-GABA (Tocris) was recirculated using a fluid pump (Cole-Parmer, IL). RuBi-GABA at this concentration does not alter membrane resistance of the cell^20^. GABA was uncaged by a brief (5 ms) illumination from a 470 nm light-emitting diode (LED) (CoolLED, Andover, UK) at 20% intensity (resulting in ~4 μM free GABA^19^) to preferentially activate high-affinity extrasynaptic GABA_A_Rs. We used a 30 second inter-sweep interval to allow for clearance of GABA between sweeps. For GABA dose-response experiments a 473 nm laser light source (PSU-III-LED, Opto Engine LLC, Midvale, UT) was used for GABA uncaging. The laser power was modulated over a range of intensities (0.28 µW–315 µW) to modulate the amount of GABA released. The maximum GABA concentration in these experiments was not more than 60 μM (the concentration of RuBi-GABA in the bath) and likely much less (20–40 μM). For this reason the saturation of responses at high laser powers likely represents saturation of GABA uncaging rather than saturation of GABA_A_ receptors. The uncaging laser intensity was measured using a photometer placed under the objective of the microscope. For each experiment uncaging current amplitudes were first measured over 3–5 minutes to obtain a stable baseline amplitude before adding CGP55845 or other drugs. Cells in which the uncaging current amplitude was not stable over this period were not included for further analysis. The tonic GABA_A_ current in granule cells was measured by the net change in holding current following application of 20 µM bicuculline (Tocris, MN). In tonic current recordings the ACSF contained 10 µM NBQX and 10 µM CPP. Access resistances of all cells were monitored and cells deviating from 20 ± 10 MΩs were discarded.

Where indicated, ACSF contained one or more of the following: 3 µM CGP 55845 (CGP, Abcam, Cambridge, MA, USA); 200 µM picrotoxin (PTX; Abcam); 200 µM 2-hydroxysaclofen (Tocris); 20 µM Bicuculline (Tocris), 100 µM Baclofen (Abcam), 10 µM NN711, 1 mM GDP-β-S (Sigma), 10 µM Forskolin (sigma), 100 µM SQ-22536 (Tocris), 1 µM PKI 14–22, 1 µM GF-109203 × , 3–6 µM Nifedipine (Tocris), 2 µM KN-62 (Tocris), 20 µM EGTA-AM (Tocris), or 10 µM Dantrolene (Tocris).

### Data analysis

Data were analyzed using IgorPro (Wavemetrics, Lake Oswego, OR, USA) using the Neuromatic plug-in^85^ and Excel (Microsoft, Redmond, WA, USA). Statistical significance was determined using 2-tailed paired Student’s t-tests in Excel (Microsoft, Redmond, WA, USA) or Prism 6 (GraphPad Software, La Jolla, CA, USA). Statistical values of p ≤ 0.05 were considered significant.

Power-spectra analysis: In order to measure the power-spectra of GABA_A_R mediated currents, GABA was applied to cerebellar granule cells by prolonged uncaging of 60 μM RuBi-GABA (12 second pulse from 473 nM LED). GABA uncaging sweeps were interleaved with background sweeps in which the LED was not activated. GABA_A_R mediated currents were first recorded in standard ACSF and then following bath application of 3 µM CGP55845. For each sweep, the power spectra was computed by fast Fourier transform over 16 segments of 8192 points each and averaged. In order to avoid time dependent changes in current amplitude, the time segments analyzed began at 5 seconds after the onset of GABA uncaging where the macroscopic GABA current was relatively stable. The power spectra of interleaved background sweeps (lacking GABA uncaging) were computed by the same method and subtracted from GABA power spectra. The power spectra was fitted with the sum of three Lorentzian functions of the form:

$${\rm{L}}({\rm{f}})={\rm{S}}/({\rm{1}}+{(2{\rm{\pi }}\ast {\rm{f}}\ast {\rm{\tau }})}^{{\rm{2}}})$$

where L(f) is the spectral density at frequency f, S is the power of the spectrum at f = 0, and τ is the time constant.

Non-stationary fluctuation analysis: GABA currents were recorded in cerebellar granule cells following either a brief (5 ms) puff of muscimol (10 μM) at the soma or uncaging of RuBi-GABA (60 μM) using a brief (5 ms) flash from a 473 nm laser. For each sweep, amplitude and variance were measured from the peak current to the end of the decay. The average current was scaled to the peak of each individual sweep and subtracted from the current trace, producing a difference trace for each sweep which was then squared to produce a variance trace. Each sweep was then divided into 30 equivalent amplitude bins and the mean current amplitude and variance were measured for each bin. The mean current was plotted against the mean variance and fitted with the following function:

$${{\rm{\sigma }}}^{{\rm{2}}}={i}{{\rm{I}}}_{{\rm{m}}}-{{{\rm{I}}}_{{\rm{m}}}}^{{\rm{2}}}/{\rm{N}}$$

where σ^2^ us the variance, I_m_ is the mean current, *i* is the single channel current, and N is the number of channels participating in the current. For all cells included in the final data set the peak current was at least 60% (mean: 78.2 ± 5.2%) of the theoretical maximal current (the x-intercept in the variance equation above). The maximum open probability (P_O max_) was calculated by dividing the peak current by (i*N), the x-intercept of the fitting function^86^.

### Modelling

GABA_A_R-mediated currents evoked by GABA uncaging in cerebellar granule cells were modelled in NEURON^87^, based on a kinetic scheme first described by Jones and Westbrook^35^, consisting of single- and double-liganded closed, open, and desensitized states. The model was adapted from the granule cell model developed by Diwakar *et al*.^88^, and was obtained from ModelDB^89^ (accession number 116835). GABA uncaging was modelled as a 5 ms square pulse of 60 μm GABA followed by an exponential decay, to replicate the relatively low concentrations of GABA used in experiments. Transition rates were optimized to fit the rise-time and decay kinetics of the recorded currents.

## Data Availability

We will make all materials, data and associated protocols derived from this work promptly available to other researches without undue qualifications or delays.
